# Costs and Projected Effect of a Federally Qualified Health Center–Based Mailed Colorectal Cancer Screening Program in Texas

**DOI:** 10.5888/pcd21.230266

**Published:** 2024-05-02

**Authors:** Todd Olmstead, Jennifer C. Spencer, Nicole Kluz, F. Benjamin Zhan, Navkiran K. Shokar, Michael Pignone

**Affiliations:** 1 Lyndon B Johnson School of Public Affairs, The University of Texas at Austin; 2 Department of Population Health, The University of Texas at Austin Dell Medical School; 3 Department of Internal Medicine, The University of Texas at Austin Dell Medical School; 4 Livestrong Cancer Institutes, The University of Texas at Austin Dell Medical School; 5 Texas Center for Geographic Information Science, Department of Geography and Environmental Studies, Texas State University, San Marcos, Texas

## Abstract

**Introduction:**

Mailed stool testing for colorectal cancer (CRC) may improve screening uptake and reduce the incidence and mortality of CRC, especially among patients at federally qualified health centers (FQHCs). To expand screening programs it is important to identify cost-effective approaches.

**Methods:**

We developed a decision-analytic model to estimate the cost, effects on screening and patient outcomes (CRCs detected, CRCs prevented, CRC deaths prevented), and cost-effectiveness of implementing a state-wide mailed stool testing program over 5 years among unscreened, age-eligible (aged 50–75 y) patients at FQHCs in Texas. We compared various outreach strategies and organizational structures (centralized, regional, or a hybrid). We used data from our existing regional mailed stool testing program and recent systematic reviews to set parameters for the model. Costs included start-up and ongoing activities and were estimated in 2022 US dollars from the perspective of a hypothetical third-party payer. Cost-effectiveness was assessed by using both incremental and average cost-effectiveness ratios.

**Results:**

Using either a statewide centralized or hybrid organizational configuration to mail stool tests to newly eligible FQHC patients and patients who have responded at least once since program inception is likely to result in the best use of resources over 5 years, enabling more than 110,000 additional screens, detecting an incremental 181 to 194 CRCs, preventing 91 to 98 CRCs, and averting 46 to 50 CRC deaths, at a cost of $10 million to $11 million compared with no program.

**Conclusions:**

A statewide mailed stool testing program for FQHC patients can be implemented at reasonable cost with considerable effects on CRC screening outcomes, especially when its structure maximizes program efficiency while maintaining effectiveness.

**Summary**

**What is already known on this topic?**

Mailed stool testing is effective for increasing colorectal cancer (CRC) screening in federally qualified heath centers (FQHCs), but evidence for efficiently scaling these programs is limited.

**What is added by this report?**

We modeled the effect and cost of implementing a state-wide mailed stool testing program among unscreened, age-eligible (aged 50–75 y) patients from 72 FQHC systems in Texas. We estimated that a 5-year program would cost $10 million to $11 million and result in 113,000 patients screened, 181 to 194 CRC cases detected, 91 to 98 CRC cases prevented, and 46 to 50 CRC deaths averted.

**What are the implications for public health practice?**

A state-wide mailed stool testing program for FQHC patients can be implemented at reasonable cost with considerable effect on CRC screening outcomes.

## Introduction

Colorectal cancer (CRC) screening is effective but underused (1). Modeling studies suggest that with complete adherence, stool-based screening with fecal immunochemical testing (FIT) can reduce colorectal cancer incidence by 47% to 72% and colorectal cancer mortality by 72% to 81% over the span of age-recommended screening (2–4). Cost-effectiveness analyses have shown that FIT-based screening strategies would be cost-saving relative to not screening (ie, implementation costs would be offset by downstream savings in deferred treatment costs), a threshold met by few screening programs (3).

Despite this potential, CRC screening has been incompletely implemented. Recent self-reported data from the Behavioral Risk Factor Surveillance System suggests that 70% of adults aged 50 to 75 years and 20% of adults aged 45 to 49 years are up to date with one of the recommended forms of screening, with most screening occurring through screening colonoscopy (5). Substantial inequities in up-to-date screening exist, particularly among people who have barriers to accessing care (eg, no health insurance, rural residence, transportation barriers, language discordance), and these inequities limit overall screening performance and may exacerbate disparities in cancer incidence and mortality (6).

Several interventions have been shown to be effective in increasing screening (7). In particular, mailed stool testing programs can help close gaps in screening (8–11). Mailed stool testing overcomes several barriers to care and has been implemented in a range of health systems, including many that serve socially and economically marginalized populations. Federally qualified health centers (FQHCs) are especially good sites for mailed stool testing programs because they serve large numbers of patients who are eligible for screening but unscreened.

Texas has the largest population without health insurance nationally and relatively low CRC screening rates, with substantial gaps among populations that lack health insurance. CRC screening rates at FQHCs in Texas are lower than national averages; only 35% of eligible patients were up to date in 2020 (12). Improving CRC screening in Texas FQHCs may substantially reduce inequities in screening, decrease CRC incidence and mortality, and serve as an example for other states. Texas, through its Cancer Prevention and Research Institute of Texas (CPRIT) initiative, has provided substantial funding for programs to improve screening rates in medically underserved populations, with strong initial results in individual communities and regions (13).

We developed and implemented a CPRIT-funded program of mailed stool testing in a large FQHC system in Central Texas, with promising results, including a substantial increase in the system CRC screening performance and reasonable costs per additional patient screened (14). Moreover, we showed that this program may be reducing screening inequities (15). However, our program and other CPRIT-funded efforts reach only a modest portion of unscreened patients in the state. Expanding mailed stool testing to all unscreened FQHC patients in Texas may improve screening and reduce the incidence and mortality of colorectal cancer. We sought to model the costs and potential effect of implementing a state-wide mailed FIT screening program at FQHCs in Texas, drawing on our experience and outcomes from Central Texas (14–16) and previous work that examined programs nationally and internationally (17,18) as a means of increasing CRC screening across this large and diverse state. We also sought to examine various scenarios for organization of the program, to help guide future program expansion and structure.

## Methods

We developed a decision-analytic model to estimate the effect, cost, and cost-effectiveness of implementing, over 5 years, a state-wide mailed FIT screening program among the approximately 215,000 unscreened, age-eligible (aged 50–75 y, based on screening guidelines in place during 2016–2021 [1,19]) FQHC patients currently receiving care in 1 of the 72 FQHC systems in Texas (12). We assumed our initial cohort would grow by 5% annually (and conducted sensitivity analysis of this assumption) during the 5-year time horizon. We chose the 5-year time horizon because of its policy relevance to Texas leaders considering different program options for increasing screening and because it corresponds to the length of typical program funding cycles.

We modeled 3 outreach strategies for each of 3 organizational configurations (9 scenarios in total). Our model is based on difference equations and differs from a traditional Markov model in that the transition probabilities are not time homogeneous but rather vary from year to year based on our longitudinal experience with our regional mailed FIT program (14). The model was implemented in Microsoft Excel 2019, and details of the model structure are available elsewhere (Appendix available at https://doi.org/10.26153/tsw/50269). Because this modeling study used fully deidentified secondary data, it did not require institutional review board approval.

### Intervention

We based the intervention on our successful program in Central Texas and assumed it would be identical across outreach strategies (14,15). The program in Central Texas demonstrated that mailed FIT was an effective and cost-effective population health strategy for CRC screening (Table 1). Briefly, patients received a mailing packet comprising an introductory letter in plain language, the FIT itself, easy-to-read instructions with pictures, a records-update postcard (for patients to indicate prior screening that was not documented and/or to opt out of future mailings), and a postage-paid laboratory mailer. All materials were provided in both English and Spanish.

**Table 1:** Key Costs and Effects of a Mailed FIT Screening Program in Central Texas, November 2017–July 2019^a^

Item	Value	Notes
**Direct unit costs**		
FIT mailers	$6.73	$1.46 labor ($29.22 per hour × 3 min per mailer) + $0.47 supplies (envelopes, address labels, paper, ink and printer) + $4.80 postage
Reminder letters	$1.15	$0.44 labor ($29.22 per hour × 0.91 min per letter) + $0.11 supplies (envelope, paper, ink and printer) + $0.60 postage
Automated text messages	$78.95	$39.48 × 2 h of set-up time (one-time fixed cost)
Results letters	$1.15	$0.44 labor ($29.22 per hour × 0.91 min per letter) + $0.11 supplies (envelope, paper, ink and printer) + $0.60 postage
Postcards	$3.96	$2.44 labor ($29.22 per hour × 5 min (to update EHR from returned health information cards)) + $1.52 postage
	$1,375	$275 per year × 5 years (annual fee for business reply mail)
FIT processing fee	$20	—
Update FIT results	$0.97	$29.22 × 2 min (to update EHR with FIT results)
Calls about positive test results	$3.96	$39.59 × 6 min
Navigation to colonoscopy	$9.90	$39.59 × 15 min
Colonoscopy	$1,800	—
**Rates**		
Start-up cost	10%	% of Year 1 cost (excluding cost of colonoscopies); includes establishing a data infrastructure; recruiting, hiring, and training staff; developing a fiscal system; developing colonoscopy capacity; and equipment.
Indirect cost	24%	% of Ongoing cost (excluding colonoscopies); includes contract execution, report generation, supervision, meeting oversight, supply management, data quality monitoring, and data management.
**Effects**		
Reminder letters	83%	% of Patients requiring a reminder letter
Returned FIT in initial year^b^	19%	% of Patients who return FIT in their initial year
Unspoiled FIT returns	95%	% of Returned FIT that could be processed (no mail delays or incorrect sample submissions)
Positivity rate in initial year^c^	6%	% of Returned FIT with positive test results
Navigation success rate	70%	% of Positive test results leading to colonoscopy
CRC detection rate	4.5%	% of Colonoscopies that detect CRC
Adenomas detection rate	39.0%	% of Colonoscopies that detect adenomas

Abbreviations: CRC, colorectal cancer; FIT, fecal immunochemical test.

a Source: Pignone et al (14). Costs are reported in 2022 US dollars.

b Probabilities of returning FIT in subsequent years conditional on returning FIT in previous years are available elswehere (https://doi.org/10.26153/tsw/50269).

c 5% in years 2 through 5.

Patients completed the test and returned it to the laboratory via prepaid mailer. Patients who did not complete the FIT received a text message 2½ weeks later and a reminder letter 5 weeks after the initial mail-out. Results were returned from the laboratory to the program and to the FQHC. Negative FIT results were communicated to patients via letter and entered into their electronic health record.

Positive FIT results were communicated via letter and telephone call recommending that the patient schedule a colonoscopy with the help of a bilingual patient navigator. Navigators provided additional education to the patient about positive test results, helped the patient schedule a pre-evaluation and colonoscopy, helped troubleshoot any problems that arose, and ensured that test results and any follow-up plan were communicated effectively. The navigator also connected patients who were diagnosed with cancer to treatment, at which point the program monitored treatment and follow-up adherence but did not directly provide cancer treatment services.

We assumed that all tests completed represented new incremental screening that would not have occurred by other means in the absence of the program. In our existing program and in this model, we do not send mailers or reminders in subsequent years if a patient has opted out. Patients who previously received a positive FIT result also do not receive mailed outreach and instead enter into surveillance based on the results of their follow-up colonoscopy.

### Outreach strategies

In deciding which patients to reach out to and how frequently, each of the modeled outreach strategies begins the same way: in year 1, FIT is mailed to all age-eligible and unscreened active patients in Texas FQHCs. Then, in years 2 through 5, FIT is mailed to all newly eligible, unscreened patients, plus 1) everyone we sent to previously, 2) those who returned FIT at least once previously, or 3) those who returned FIT in the previous year.

### Organizational configurations

We considered 3 options for organizing the operations of a state-wide mailed FIT program in Texas: regional, centralized, and hybrid. Because current CPRIT-funded CRC screening programs are organized locally or regionally (13), we assumed the regional organizational configuration would comprise 7 independent regions approximately the size of our Central Texas program (25,000–50,000 eligible patients), with each region responsible for its own mailing and navigation operations (Figure). In contrast, we assumed a centralized organizational structure would simultaneously serve all FQHC systems in Texas by housing all state-wide mailing and colonoscopy navigation operations under a single roof. Finally, we assumed the hybrid organizational configuration would include a centralized mailing operation, but navigation would be conducted at 7 regional navigation sites.

**Figure:**
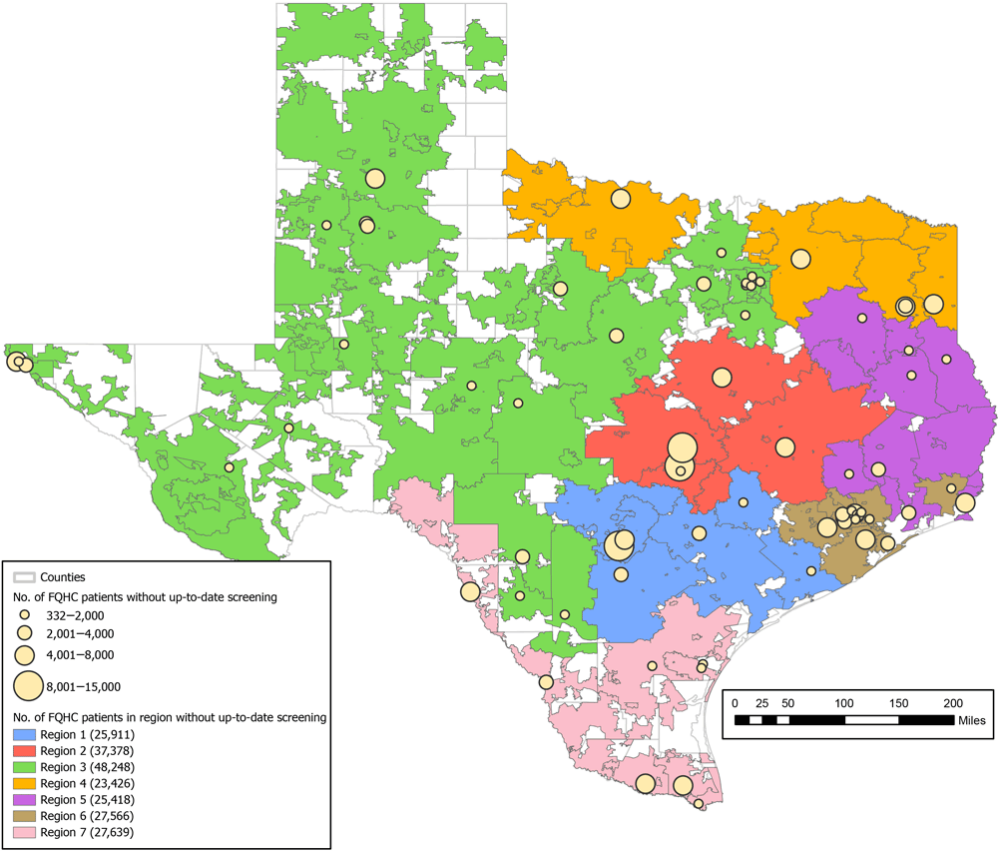
Regionalization of age-eligible patients (aged 50–74 y) without up-to-date colorectal cancer screening served by federally qualified health centers (FQHCs) in Texas in 2020. Uncolored areas were not covered by FQHCs in 2020. Circles indicate the county locations of FQHC administrative offices and are scaled to represent the number of age-eligible FQHC patients without up-to-date screening.

The base model assumptions included 6 key parameters that distinguish among the regional, centralized, and hybrid organizational configurations: indirect cost, start-up cost, unspoiled screens (spoiled screens are those that cannot be processed due to improper collection or delay), successful navigation, FIT processing fee, and navigation time (Table 2). The assumptions underlying the regional configuration were based on our Central Texas program (14), while the assumptions for the centralized and hybrid configurations were based on our Central Texas model supplemented by values from other programs (17,20–25) and expert judgement. We also performed sensitivity analyses.

**Table 2:** Base Model Assumptions, by Organizational Configuration, for a Statewide Mailed FIT Program at Federally Qualified Health Centers, Texas

Item	Centralized^a^	Hybrid^b^	Regional^c^
Indirect cost^d^	15%	20%	24%
Start-up cost^e^	5%	7.5%	10%
Unspoiled screens	90%	90%	95%
Successful navigation	65%	70%	70%
FIT processing fee	$20	$20	$23
Navigation time	18 min	15 min	15 min

Abbreviation: FIT, fecal immunochemical test.

a A centralized organizational structure would simultaneously serve all Texas FQHC systems by housing all state-wide mailing and colonoscopy navigation operations under a single roof.

b A hybrid organizational configuration would include a centralized mailing operation, but navigation would be conducted at 7 regional navigation sites.

c A regional organizational configuration would comprise 7 independent regions (25,000–50,000 eligible patients), with each region responsible for its own mailing and navigation operations.

d Indirect cost = % of total ongoing cost (excluding cost of colonoscopies).

e Start-up cost = % of year 1 total cost (excluding cost of colonoscopies).

We assumed that a centralized configuration would enjoy efficiencies through economies of scale (eg, volume discounts on FIT processing) and lower indirect and start-up costs, compared with the regional configuration, but also may have a higher spoilage rate for returned screens (due to presumably longer postal transit times) and a less effective and efficient navigation operation (due to centralized navigators presumably having less familiarity with resources in each region). We further assumed the hybrid configuration would combine the mailing advantages of a centralized operation with the navigation advantages of regional operations, in which the navigators’ rich local knowledge may increase the likelihood of achieving timely and effective colonoscopy.

### Costs

We estimated the 5-year cost, including both start-up and ongoing costs, of implementing each of the 9 scenarios. Ongoing direct costs of the intervention itself (Table 1 [14]) were assumed to be fixed across organizational configurations, with the exception of FIT processing, which was assumed highest in a regional configuration, and navigation time, which was assumed highest in a centralized configuration (Table 2).

Start-up costs were assumed to be a fixed percentage of first-year ongoing costs (excluding the cost of colonoscopies) and included activities such as establishing a data infrastructure; recruiting, hiring, and training staff; developing a fiscal system; developing colonoscopy capacity; and acquiring equipment. Ongoing indirect costs were assumed to be a fixed percentage of the total ongoing costs (excluding the cost of colonoscopies) and included activities such as contract execution, report generation, supervision, meeting oversight, supply management, data quality monitoring, and data management. The fixed percentages assumed for the start-up and ongoing indirect costs varied by organizational configuration (Table 2) (14,17,20–25).

We focused on estimating implementation costs, so we did not include the costs of cancer treatment or costs saved from cancers prevented. Costs are estimated in 2022 US dollars from the perspective of a hypothetical third-party payer (eg, CPRIT) responsible for covering program costs as well as costs of colonoscopy for patients without health insurance (39% of unscreened patients [12]). We did not discount costs or outcomes under the assumption that medical inflation would exceed general inflation such that the real rate of return multiplier would approximate unity (and conducted sensitivity analysis around this assumption) (20,26).

### Outcomes

We modeled 3 patient outcomes: CRC cases detected, CRC cases prevented, and CRC deaths prevented. Although all 3 patient outcomes are important to decision makers, for this study we focused mainly on the outcome CRC cases detected because it is based directly on inputs from our Central Texas program, whereas the 2 prevention outcomes are based, in part, on estimates in previous models that reflect the population prevalence of CRC and the sensitivity of testing (2,3,27).

We also modeled 2 process outcomes: the number of unspoiled completed FITs and the number of colonoscopies required. We estimated the incremental 5-year totals for each outcome obtained in each of the 9 scenarios.

### Cost-effectiveness

We assessed cost-effectiveness in 2 ways. First, we used traditional incremental cost-effectiveness ratios (ICERs) (26), defined in this study as the incremental cost of using a given scenario, compared with the next least costly scenario, to obtain an additional unit of outcome (eg, an additional CRC case detected). Scenarios were eliminated if they were either strictly dominated (ie, another scenario was both less costly and more effective) or weakly dominated (ie, a linear combination of 2 other scenarios was both less costly and more effective) (26,28). Of the remaining nondominated scenarios, the cost-effective scenario is the one with the largest ICER that falls below the threshold willingness-to-pay value placed by decision makers on an additional unit of outcome (29). The cost-effectiveness analysis also considered a “no program” alternative that was assumed to have zero additional cost and zero incremental effect (26).

Because program budgets are typically limited, we also assessed cost-effectiveness by using average cost-effectiveness ratios (ACERs), enabling us to identify the scenario that would provide the greatest number of a given outcome within a fixed budget (ie, the most efficient scenario). For illustrative purposes, we chose a fixed budget of $5 million because this amount is consistent with the size of a typical large CPRIT grant (13).

We followed the best practices recommended in the Consolidated Health Economic Evaluation Reporting Standards (CHEERS) reporting guidelines (30; Appendix available at https://doi.org/10.26153/tsw/50269).

### Sensitivity analysis

To test the robustness of our conclusions, we conducted 1-way sensitivity analyses on several key parameters common to all scenarios (growth rate of population of interest, percentage of returned FITs in initial year, test positivity rate, CRC detection rate, direct cost, percentage of FQHC patients without health insurance, discount rate), varying each of these parameters by a minimum of +/−20% from their base values. We then conducted threshold sensitivity analyses on the 6 key model parameters (Table 2) that varied across organizational configurations.

## Results

Outreach only to patients who had returned a FIT in the previous year was always the least expensive option ($7.8 million–$9.4 million), followed by outreach to patients who had returned a FIT at least once previously ($9.9 million–$11.9 million) (Table 3). Additional tests completed ranged from 85,000 to 140,000 across scenarios and required 3,100 to 5,400 additional colonoscopies.

**Table 3:** Five-Year Program Cost, Five-Year Incremental Outcomes, and ICERs, by Scenario, for a Statewide Mailed FIT Program at Federally Qualified Health Centers, Texas

Scenario	Cost, in millions, $	Incremental outcomes					ICER, in thousands, $		
		CRC cases detected	CRC cases prevented	CRC deaths prevented	Screens completed, in thousands	Colonoscopies, in thousands	CRC cases detected	CRC cases prevented	CRC deaths prevented
**No program**	0	0	0	0	0	0	—	—	—
**Patients who returned FIT in the previous year**									
Centralized^a^^,^ b	7.8	140	69	35	85.3	3.1	WD	WD	WD
Hybrid^b^^,^ c	8.4	150	74	37	85.3	3.3	WD	WD	WD
Regional^b^^,^ d	9.4	158	78	39	89.8	3.5	WD	WD	WD
**Patients who returned FIT at least once previously**									
Centralized^a^^,^ b	9.9	181	91	46	113.1	4.0	WD	WD	WD
Hybrid^c^	10.6	194	98	50	113.1	4.3	54.8	108.3	214.9
Regional^d^	11.9	204	104	52	119.0	4.5	130.4	249.6	495.6
**All patients who were ever sent a FIT**									
Centralized^a^^,^ e	17.5	215	107	54	132.0	4.8	WD	WD	WD
Hybrid^c^^,^ e	18.7	232	115	58	132.0	5.1	WD	WD	WD
Regional^d^	20.5	244	121	61	139.3	5.4	217.2	479.7	952.3

Abbreviations: CRC, colorectal cancer; FIT, fecal immunochemical test; ICER, incremental cost-effectiveness ratio; WD, weakly dominated.

a A centralized organizational structure would simultaneously serve all Texas FQHC systems by housing all state-wide mailing and colonoscopy navigation operations under a single roof.

b This scenario is weakly dominated for all patient outcomes by the combination of “no program” and “at least once/hybrid.”

c A hybrid organizational configuration would include a centralized mailing operation, but navigation would be conducted at 7 regional navigation sites.

d A regional organizational configuration would comprise 7 independent regions (25,000–50,000 eligible patients), with each region responsible for its own mailing and navigation operations.

e This scenario is weakly dominated for all patient outcomes by the combination of “at least once/regional” and “everyone/regional.”

Outreach to the population who had returned a FIT at least once previously combined with use of a hybrid configuration weakly dominated (along with “no program”) all lower-cost statewide options and identified an additional 194 CRC cases in the population at an ICER of $54,800 per CRC case detected compared with no program. Outreach to the population who had returned a FIT at least once previously combined with the use of a regional configuration cost an additional $1.3 million more than the hybrid configuration but identified an additional 10 CRC cases, for an ICER of $130,400 per CRC case detected. Finally, outreach to all patients who were ever sent a FIT (ie, everyone in every year) is the most expensive ($17.5 million–$20.5 million) and the most effective (215–244 CRC cases detected). The “everyone”/regional option weakly dominated (along with the “at least once”/regional option) the “everyone”/hybrid and “everyone”/centralized options and resulted in an ICER of $217,200 per CRC case detected compared with the “at least once”/regional option.

In our calculation of what each scenario could achieve with an investment of $5 million, 2 patterns emerged (Table 4). First, if we fixed the organizational configuration, the outreach strategy to the population who had returned a FIT at least once previously was always more efficient at achieving every outcome than either the “everyone” strategy or the “previous year” strategy. As a practical matter, compared with “at least once” scenarios, the “previous year” strategy reduced the reach very quickly over time, thereby making it difficult to justify the start-up costs; and because fewer than 3% of FQHC patients who do not respond in year 1 do so in year 2 (14), the “everyone” scenarios were inefficient overall. Second, if we fixed the outreach strategy, we found that differences between centralized and hybrid configurations were small, but both were more efficient at achieving every outcome compared with a regional configuration. Our base results suggest that under a constrained budget, the “at least once”/hybrid scenario is preferred, and the “at least once”/centralized scenario is a close second.

**Table 4:** Five-Year Incremental Outcomes Under a Fixed Budget of $5 Million, and ACERs, by Scenario, for a Statewide Mailed FIT Program at Federally Qualified Health Centers, Texas

Scenario	Incremental outcomes					ACERs, in 1,000s, $		
	CRC cases detected	CRC cases prevented	CRC deaths prevented	Screens, in thousands	Colonoscopies, in thousands	CRC cases detected	CRC cases prevented	CRC deaths prevented
**Patients who returned FIT in the previous year**								
Centralized^a^	89.6	44.1	22.2	54.8	1.99	55.8	113.4	225.0
Hybrid^b^	89.6	44.2	22.2	50.9	1.99	55.8	113.2	224.8
Regional^c^	83.8	41.4	20.9	47.7	1.86	59.6	120.8	239.8
**Patients who returned FIT at least once previously**								
Centralized^a^	91.1	46.0	23.2	57.0	2.02	54.9	108.6	215.7
Hybrid^b^	91.2	46.2	23.3	53.1	2.03	54.8	108.3	214.9
Regional^c^	85.5	43.3	21.8	49.8	1.90	58.5	115.4	229.0
**All patients who were ever sent a FIT**								
Centralized^a^	61.6	30.6	15.4	37.8	1.37	81.2	163.5	324.7
Hybrid^b^	62.0	30.8	15.5	35.3	1.38	80.6	162.4	322.4
Regional^c^	59.4	29.6	14.9	33.9	1.32	84.2	169.0	335.5

Abbreviations: ACER, average cost-effectiveness ratio; CRC, colorectal cancer.

a A centralized organizational structure would simultaneously serve all Texas FQHC systems by housing all state-wide mailing and colonoscopy navigation operations under a single roof.

b A hybrid organizational configuration would include a centralized mailing operation, but navigation would be conducted at 7 regional navigation sites.

c A regional organizational configuration would comprise 7 independent regions (25,000–50,000 eligible patients), with each region responsible for its own mailing and navigation operations.

### Sensitivity analysis

One-way sensitivity analysis on key parameters common to all scenarios showed that the rank ordering of the ICERs and ACERs in our base results did not change across plausible ranges of most parameters (Appendix available at https://doi.org/10.26153/tsw/50269).

Given that program budgets are typically limited (so efficiency matters) and the 1-way sensitivity analysis results showed that each “at least once” scenario was consistently preferred to (was more efficient than) the other outreach strategies, we next assessed the relative efficiency of the 3 “at least once” scenarios by using threshold sensitivity analysis on the key parameters that varied by organizational configuration (Table 2).

With the “at least once” outreach strategy, we found that the hybrid organizational configuration remained more efficient (had a lower ACER) than the regional configuration across all reasonable threshold values of our key parameters. For example, the hybrid organizational configuration remained more efficient than the regional organizational configuration in detecting CRCs until the indirect cost rate of the hybrid configuration was greater than that for regional (26.9% vs 24.0%), which is logically impossible. Thus, we are confident that the hybrid configuration is preferred to (more efficient than) regional configuration for detecting CRCs.

In contrast, each of the following slight-to-moderate changes in the parameters of a centralized program would make it as efficient as the hybrid configuration in detecting CRCs: 1) increasing the successful navigation rate from 65.0% to 65.15% (a 0.2% increase), 2) decreasing the indirect cost rate from 15.0% to 14.8% (a 1.3% reduction), or 3) decreasing the start-up cost rate from 5.0% to 4.5% (a 10.0% reduction). Moreover, if centralized navigators were as successful and timely as regionalized navigators (as used in a hybrid program), then average cost per CRC case detected in a centralized configuration would decrease to $52,005, thereby making it more efficient than a hybrid configuration ($54,800).

## Discussion

This modeling study projected the effect, cost, and cost-effectiveness of 9 scenarios for implementing a state-wide mailed FIT screening program among the more than 200,000 unscreened, age-eligible FQHC patients in Texas.

Under a traditional cost-effectiveness framework, we found several alternatives that decision makers should consider based on willingness to pay for improving CRC outcomes. Notably, if decision makers are willing to pay at least $217,200 per additional CRC case detected or nearly $1 million per CRC death averted, our model recommends combining the “everyone” outreach strategy (ie, mailing to everyone each year despite previous adherence) with the regional organizational configuration, because this scenario results in the most favorable outcomes, albeit at the highest cost. At a lower willingness-to-pay threshold ($54,800 per CRC case detected or $214,900 per CRC death prevented), decision makers should consider combining the “at least once” strategy (ie, ongoing mailing to anyone who has participated once) with a hybrid configuration. These results are robust to extensive sensitivity analyses.

In contrast, if program funding is budget-constrained, which would likely be the situation for a state-wide mailed FIT screening program, then decision makers should consider combining the “at least once” outreach strategy with the hybrid organizational configuration, because this scenario results in the greatest improvement in outcomes within a fixed budget. However, this recommendation is highly sensitive to the relative effectiveness of centralized versus regional navigators. Simply put, if centralized navigators can be trained to be as effective as regional navigators, then the centralized configuration would be the strongly preferred organizational configuration. Thus, our study raises an important empirical question about the relative effectiveness of centralized versus regional navigation operations.

Taken together, our findings suggest that the “at least once” outreach strategy combined with a hybrid organization configuration would likely be the best value (followed closely by the “at least once” strategy combined with a centralized configuration), because it is both the most efficient scenario overall and cost-effective over a wide range of willingness-to-pay values for an additional CRC detected.

Our study provides actionable, state-level information on implementation costs for a region with large numbers of uninsured and unscreened FQHC patients. Previous analyses have found implementing mailed FIT screening in FQHC settings to be highly cost-effective (22). Our results complement modeling that found that a national mailed program (with mailing to all age-eligible adults) would result in 8.7 million people screened at a cost of $318 million per year, preventing 2,900 CRC deaths each year (17). National-level analyses from other countries have found similar results (18), as has a modeling study focused on Oregon Medicaid beneficiaries (31).

### Strengths and limitations

Strengths of our study include modeling a wide range of scenarios for implementing a state-wide mailed FIT screening program and the use of real-world cost and effectiveness data from our existing program in a Central Texas FQHC (albeit not from a randomized controlled trial) (14). For each scenario, we estimated the 5-year program costs, including ongoing (direct and indirect) and start-up costs, and various patient and process outcomes. We also conducted extensive sensitivity analyses to test the robustness of our findings.

Our analysis has several limitations. We did not include patient costs associated with adherence, nor did we include costs downstream of colonoscopies (eg, costs of cancer treatment, costs saved from cancers prevented, costs of adverse events associated with colonoscopies). We assumed the modeled scenarios would use the same intervention as in our successful Central Texas program, and so the projected costs and effects might have been somewhat different had we assumed an alternative base intervention. Nevertheless, the cost-effectiveness of our intervention compares favorably with other mailed FIT programs reported in the literature (22–25), and we think it unlikely that the rank ordering of the scenarios would change under an alternative base intervention. Finally, although our Central Texas data may not be generalizable to the state, other programs in different parts of Texas have also been successful with similar models of care (16).

### Conclusion

We project that a 5-year state-wide mailed FIT screening program among FQHC patients in Texas that follows our recommended outreach strategy (ongoing mailing to anyone who has participated once) in combination with either a hybrid or centralized organizational configuration would enable more than 110,000 additional screens, detect an incremental 181 to 194 CRCs, prevent 91 to 98 CRC cases, and prevent 46 to 50 CRC deaths, at an implementation cost of $10 million to $11 million compared with the status quo (and not accounting for the savings of reduced treatment costs). Future comparative effectiveness research should ascertain the relative effectiveness of navigation in centralized and hybrid organizational configurations to help guide the optimal organization of such a program.
